# Domestic Correspondence

**Published:** 1890-06

**Authors:** C. Stoddard Smith

**Affiliations:** Secretary; Chicago, Ill.


### Domestic Correspondence.

To the Editor:

*Illinois State Board of Dental Examiners.—A Correction.*—At a meeting of this Board, held at Springfield, May 15 last, attention was called to a statement in the May number of this Journal (p. 319),[1] to the effect that this Board had conducted the final examinations of the graduating classes of the University Dental College. This not being the case, the secretary was instructed to inform the editor as to the facts, and to deny the implied statements.

The facts are that the faculty of the college named did invite the Board, through its secretary, to conduct their examinations. The secretary and Dr. Koch, who constitute a committee to examine into the working of the colleges of this State, declined most positively to do so. They consented, however, to be present as spectators, as a committee of the Board, for the purpose of gaining information, and were present for that purpose only. The Board ratified the action of the committee by declining to conduct any examinations, and informed the college that they would be present only as spectators for the above purpose when convenient.

C. Stoddard Smith,
*Secretary.*

Chicago, Ill.

---

[1] The "item" referred to in the above letter was forwarded to us by our regular Chicago correspondent, and was intended for the April issue. It read, "By a vote of the faculty and also of the graduating class, the Illinois State Board of Dental Examiners *have been* requested to conduct the final examinations of the graduating class. The examinations of the State Board *will be* an oral in addition to the regular written examinations conducted by the Faculty." It did not reach us in time, however, for the April issue, and as the examinations were set for the 29th of April, and as we did not hear from our correspondent that the examinations had not taken place, as announced, we took the liberty of changing the tense and making what had been an announcement an accomplished fact. We fail to see any just reason why any State Board should refuse to conduct the examination of any institution when requested so to do. It marks a step in advance and should be heralded with pleasure. The college, however, should set aside a portion of the examination fees to meet the expenses of the Board. Speed the day when the final examinations of all our colleges shall be conducted by State or National Boards. Then will the power granted to certain institutions under the so-called "vested rights" be minimized and dental education take the stand it should.—Ed.

